# Women’s Empowerment as It Relates to Attitudes Towards and Practice of Female Genital Mutilation/Cutting of Daughters: An Ecological Analysis of Demographic and Health Surveys From 12 African Countries

**DOI:** 10.3389/fsoc.2021.685329

**Published:** 2022-01-14

**Authors:** Carolina V. N. Coll, Thiago M. Santos, Andrea Wendt, Franciele Hellwig, Fernanda Ewerling, Aluisio J. D. Barros

**Affiliations:** ^1^ International Center for Equity in Health, Federal University of Pelotas, Pelotas, Brazil; ^2^ Postgraduate Program in Epidemiology, Federal University of Pelotas, Pelotas, Brazil

**Keywords:** empowerment, gender equality, social norms and community influence, female genital muitilation, inequalities, violence against women

## Abstract

**Background:** Women’s empowerment may play a role in shaping attitudes towards female genital mutilation/cutting (FGM/C) practices. We aimed to investigate how empowerment may affect women’s intention to perpetuate FGM/C and the practice of FGM/C on their daughters in African countries.

**Materials and methods:** We used data from Demographic and Health Surveys carried out from 2010 to 2018. The countries included in our study were Benin, Burkina Faso, Chad, Côte d´Ivoire, Ethiopia, Guinea, Kenya, Mali, Nigeria, Senegal, Tanzania, and Togo. This study included 77,191 women aged 15–49 years with at least one daughter between zero and 14 years of age. The proportion of women who reported having at least one daughter who had undergone FGM/C as well as the mother’s opinion towards FGM/C continuation were stratified by empowerment levels in three different domains (decision-making, attitude to violence, and social independence) for each country. We also performed double stratification to investigate how the interaction between both indicators would affect daughter’s FGM/C.

**Results:** The prevalence of women who had at least one daughter who had undergone FGM/C was consistently higher among low empowered women. Tanzania, Benin, and Togo were exceptions for which no differences in having at least one daughter subjected to FGM/C was found for any of the three domains of women’s empowerment. In most countries, the double stratification pointed to a lower proportion of daughters’ FGM/C among women who reported being opposed to the continuation of FGM/C and had a high empowerment level while a higher proportion was observed among women who reported being in favor of the continuation of FGM/C and had a low empowerment level. This pattern was particularly evident for the social independence domain of empowerment. In a few countries, however, a higher empowerment level coupled to a favorable opinion towards FGM/C was related to a higher proportion of daughters’ FGM/C.

**Conclusion:** Women’s empowerment and opinion towards FGM/C seems to be important factors related to the practice of FGM/C in daughters. Strategies to improve women’s empowerment combined with shifts in the wider norms that support FGM/C may be important for achieving significant reductions in the practice.

## Background

Female genital mutilation/cutting (FGM/C)- defined by “female genital mutilation” by the World Health Organization and also known as “female circumcision” among practicing communities, is a ritual practice that involves the partial or total removal of the external genitalia and/or injury to the female organs without medical justification and consent (WHO, 1997; Abdulcadir et al., 2019; Abdulcadir et al., 2021). Like any form of genital cutting, FGM/C risks surgical and other health-related complications; however, the specific risks involved and whether there are long-term adverse effects depends on the specific type of FGM/C performed and the circumstances of its performance (e.g., whether it has been medicalized or carried out in a more traditional manner) (Abdulcadir et al., 2021). Despite socio-cultural variations in FGM/C across countries, the practice is widely considered by cultural outsiders and/or opponents of the practice a harmful traditional custom that violates women and girls’ rights to health and wellbeing (WHO, 2010; Earp and Johnsdotter, 2021), which is why various agencies and campaigners, including the United Nations, aim to eliminate non-Western associated FGM/C by 2030 (Nations, 2015). FGM/C has been reported to occur worldwide, but the practice is most prevalent in the Western, Eastern, and North-Eastern regions of Africa, in parts of South and Southeast Asia, and among migrants from these areas (WHO, 2008). While prevalence rates of FGM/C have been decreasing over time in many countries in the last years, changes have been more slowly in others and socioeconomic differentials in FGM/C have grown (WHO, 2008; Shell-Duncan, 2020), particularly in countries with moderate-to-higher overall prevalence where the practice is likely to be more entrenched (Batyra et al., 2020).

FGM/C is deeply rooted in cultural beliefs and social norms perpetuated over generations which are related to ideals of femininity and modesty including the notion that FGM/C will preserve virginity and reduce promiscuity, increasing girls’ marriageability (WHO, 2020). The decision-making process to perform FGM/C is therefore influenced by social norms and community beliefs and usually lies within the confines of households, involving more than one individual of the family—each one with different power over the decision (Alradie-Mohamed et al., 2020; Cappa et al., 2020). In most countries, mother, grandmothers, and other women (e.g., aunties) are the key decision-makers with fathers and other men playing a limited role in the decision-making process (Alradie-Mohamed et al., 2020).

Women’s empowerment—defined as the gain of agency and increase of women’s autonomy can be associated with FGM/C dynamics, including judgments and choices for their daughters. In some contexts, for example, in Egypt, more empowered women (as measured by a sum of questions about decision-making)were less likely to believe that FGM/C should be continued (Afifi, 2009; Van Rossem et al., 2015). Women with higher social position, measured by their educational and literacy levels as well possession of household assets, were more likely to be against the practice of FGM/C for their daughters than low-empowered low-educated women (Van Rossem et al., 2015). In the same line, a study in the Iraqi Kurdistan region showed that the occurrence of FGM/C in daughters was negatively associated with educational level and employment status (Shabila, 2017). In addition, a qualitative study in Ghana identified the lack of young women’s autonomy, translated as the need to conform to traditional requirements and social pressure, as a driver for FGM/C continuing in the country (Sakeah et al., 2019).

Taken together, the available evidence suggest that more empowered women are less likely to have a favorable opinion regarding FGM/C and, consequently, less likely to have their daughters undergoing the procedure. Analyses of double stratification are gaining importance and visibility to identify subgroups of the population that may require stronger efforts to achieve progress (Larson et al., 2016). However, few studies have investigated the FGM/C combining aspects such opinion of woman (sometimes defined by social norms) and level of empowerment of this woman. We aimed to investigate how mother’s opinion on whether FGM/C should be continued and the practice of FGM/C among their daughters vary according to women’s empowerment levels in 12 African countries. Additionally, we evaluate how the interaction between women’s empowerment and their opinion about the continuation of practice correlated with daughters’ FGM/C by performing double stratification according to the different subgroups of these variables.

## Methods

We used data from Demographic and Health Surveys (DHS), which are nationally representative surveys that aim to assess and monitor health and nutrition of women and their children (USAID, 2021). Some of the DHS collected detailed information on FGM/C including whether the women and their daughters had undergone the procedure, their attitudes, and beliefs towards the practice. For this study, we selected surveys carried out since 2010 with data on both women’s empowerment and FGM/C. Twelve countries were included: Benin, Burkina Faso, Chad, Côte d´Ivoire, Guinea, Mali, Nigeria, Senegal and Togo from West and Central Africa, and Ethiopia, Kenya and Tanzania from Eastern and Southern Africa. Survey years ranged from 2010 to 2018. The study sample included women aged 15–49 years who were married or in a union with at least one daughter between zero and 14 years of age. Supplementary Table S1 shows survey years and sample sizes for the study countries.

### Measures

Our main outcome was whether women had at least one daughter aged less than 15 years who had undergone some form of ritual FGM/C. DHS surveys have a module about FGM/C with questions for each daughter under 15-year-old. The question used to measure FGM/C in this study was the following: “Is (NAME OF DAUGHTER) circumcised?”

Two main socially relevant variables around FGM/C were analyzed: women’s empowerment and opinion regarding the practice of FGM/C.

Women’s empowerment was measured using the SWPER Global, a survey-based indicator of women’s empowerment. The SWPER was previously validated to be used across African countries and, more recently, expanded to all low and middle-income countries (Ewerling et al., 2017; Ewerling et al., 2020a). The external validity assessment of the index showed overall strong correlations between all dimensions of the SWPER with their rankings in the Gender Development Index, a widely used indicator that captures gender inequality at the country level (Ewerling et al., 2017). Construct validity of the SWPER was also previously assessed at the individual level, through its association with modern contraceptive use, institutional delivery, stunting (Ewerling et al., 2017) and, more recently, with child developmental outcomes (Ewerling et al., 2020b) and neonatal, infant and under-5 mortality (Doku et al., 2020). The SWPER index is based on 14 questions, allowing the assessment of three empowerment domains independently as proposed by the authors attitude to violence, which comprises questions related to the women’s opinion on whether beating the wife is justified in some situations social independence, that includes the woman’s access to information, educational attainment, age at first marriage and first child, and difference in age and education between the woman and her husband; and decision making which comprises three questions on who makes decisions in the household in regard to the respondent’s health care, major expenses and visits to family and relatives. Women were categorized as having low, medium, or high empowerment level using the cut-offs provided with the SWPER Global methodology, based on the statistical distribution of the scores in each domain (Ewerling et al., 2020a). In the social independence domain, which presents a normal distribution, the score was divided into terciles while the multimodal distribution of the violence and decision-making domains was considered to define the cut-offs. The SWPER is available only for married women. Full details on the construction of the index, its categorization and validity are presented elsewhere (Ewerling et al., 2020b).

The women’s opinion on the continuation of FGM/C was evaluated with the question “Do you think that female circumcision should be continued, or should it be stopped?” The response options were the following: “continued,” “stopped,” “depends” and “don’t know.” Women who answered “continued” were classified as being in favor of FGM/C while women who answered “stopped” were classified as against FGM/C. Those who answered “depends” or “don’t know” were excluded from main analysis because these categories did not express a clear position regarding FGM/C and because the number of women in these categories were too small for most of countries. The results for these categories are available for consultation in the Supplementary Tables S8–S10.

### Statistics

We calculated the proportion of women with at least one circumcised daughter and its respective 95% confidence intervals (95%CI) in each country. These estimates as well as the proportion of women with favorable opinion on the continuation of FGM/C were stratified by women’s empowerment level. Finally, we explored the double stratification of the women’s opinion on FGM/C and the women’s empowerment level in relation to the daughters’ FGM/C by calculating its proportion for each combination of opinion category and empowerment level (Larson et al., 2016). We repeated those analyses for each domain of women’s empowerment (attitude to violence, social independence, and decision making). We presented all stratified results using equiplots (https://www.equidade.org/equiplot). Results for subgroups with sample size smaller than 25 women were suppressed from the tables and figures, in accordance with the standard practice of DHS reporting (Croft, 2018) Statistical analyses were performed using Stata 16 (StataCorp. 2019. Stata Statistical Software: Release 16. College Station, TX: StataCorp LLC). All analyses took into account the complex survey design by implementing the sample weights. The plots were created using the software R, version 4.0.2.

## Results

**FIGURE 1:**
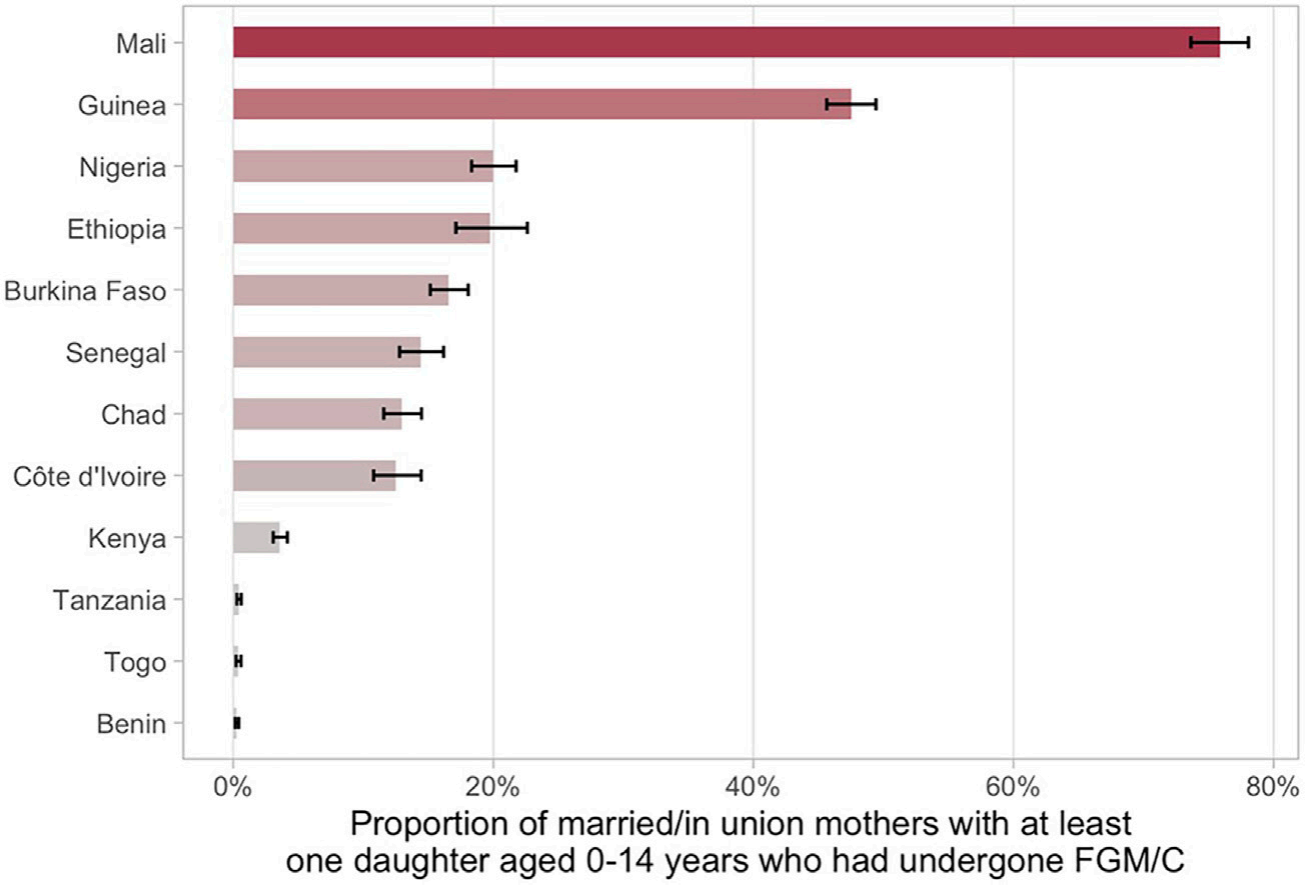
Proportion of married/in union mothers with at least one daughter aged 0–14 years who had undergone FGM/C and 95% confidence intervals.

This study included data from 12 countries, totaling 77,191 women with at least one daughter aged 0–14 years. The proportion of women with at least one daughter who had undergone FGM/C ranged from 0.3% in Benin (95% CI: 0.2–0.4) to 75.9% in Mali (95% CI: 73.6–78.1) (Figure 1).

### FGM/C Opinion And Daughter’s FGM/C by Women’s Empowerment Levels

**FIGURE 2:**
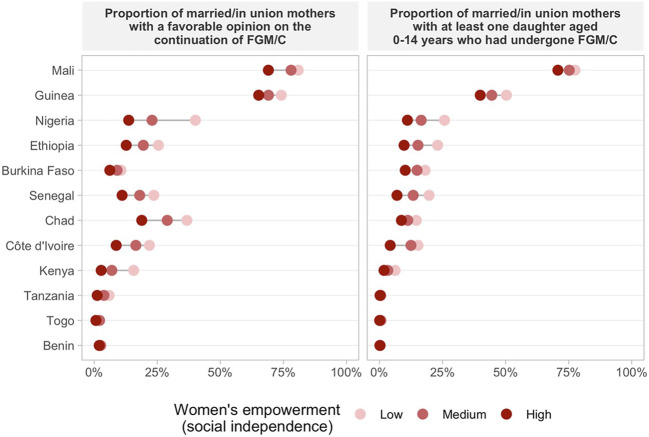
Proportion of married/in union mothers with a favorable opinion on the continuation of FGM/C and at least one daughter aged 0–14 years who had undergone FGM/C by women’s empowerment level on social independence.

**FIGURE 3:**
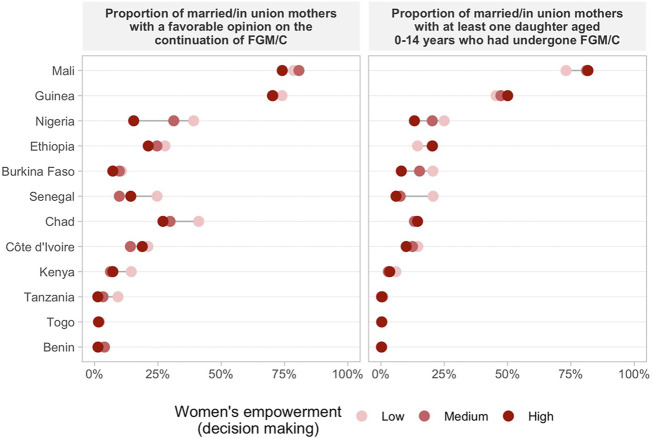
Proportion of married/in union mothers with a favorable opinion on the continuation of FGM/C and at least one daughter aged 0–14 years who had undergone GFM/C by women’s empowerment level on decision making.

**FIGURE 4:**
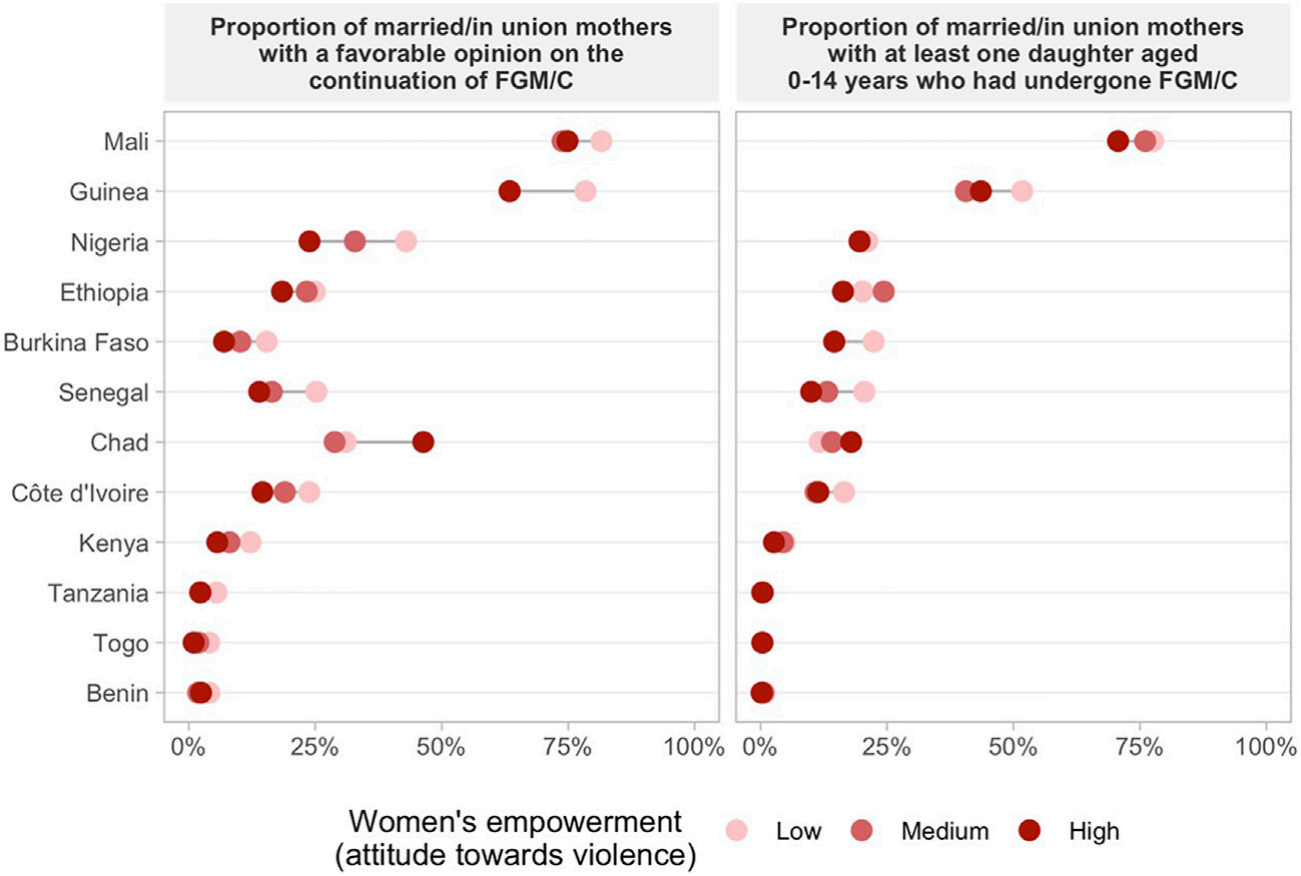
Proportion of married/in union mothers with a favorable opinion on the continuation of FGM/C and at least one daughter aged 0–14 years who had undergone GFM/C by women’s empowerment level on attitude towards violence.

Regarding women’s opinion on whether the practice of FGM/C should be continued or stopped according to the empowerment level, we found consistent positive associations across countries indicating that more empowered women are more frequently against FGM/C for all domains of the SWPER (Figures 2–4 and Supplementary Tables S2–S4). One exception was Chad, in which a higher proportion of women with a favorable opinion towards FGM/C was found among women with higher empowerment levels for the attitude towards violence domain, 31.0% (95% CI: 27.4–34.9%) of the low empowered women were in favor against 46.4% (95% CI: 39.7–53.1%) of the highly empowered. For all domains of empowerment, the largest prevalence gap between low and high empowered women was found for Nigeria: in the social independence domain; 40.1% (95% CI: 37.2–43.1%) of the low empowered women were in favor of stopping FGM/C, while among the highly empowered women this percentage was 13.7% (95% CI: 11.9–15.7%). These proportions were 39.1% (95% CI: 36.1–42.2%) and 15.4% (95% CI: 13.4–17.7%) for the decision-making domain and 42.9% (95% CI: 39.4–46.6%) and 23.9% (95% CI: 21.7–26.2%) for the attitude to violence domain.

The prevalence of at least one daughter who had undergone FGM/C was also consistently higher among low empowered women in comparison to those with medium or high empowerment levels in most countries (Figures 2–4 and Supplementary Tables S5–S7). Tanzania, Benin, and Togo were exceptions for which no differences between the groups were observed in the three domains of empowerment, but the prevalence of FGM/C was very low in these countries. Additionally, no differences on the outcome by women’s empowerment level were observed for Chad (decision-making), Nigeria and Kenya (attitude to violence).

### Double Stratification of Women’s Empowerment and Opinion Towards the Continuation of FGM/C

**FIGURE 5:**
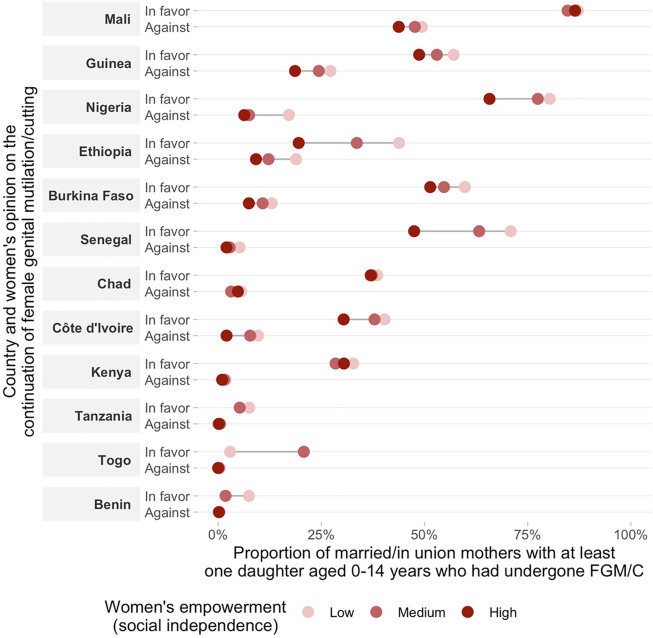
Double stratification of the daughters’ FGM/C according to the mothers’ attitudes towards FGM/C and women’s empowerment (social independence domain). Results for subgroups with sample size smaller than 25 were suppressed from the figure.

**FIGURE 6:**
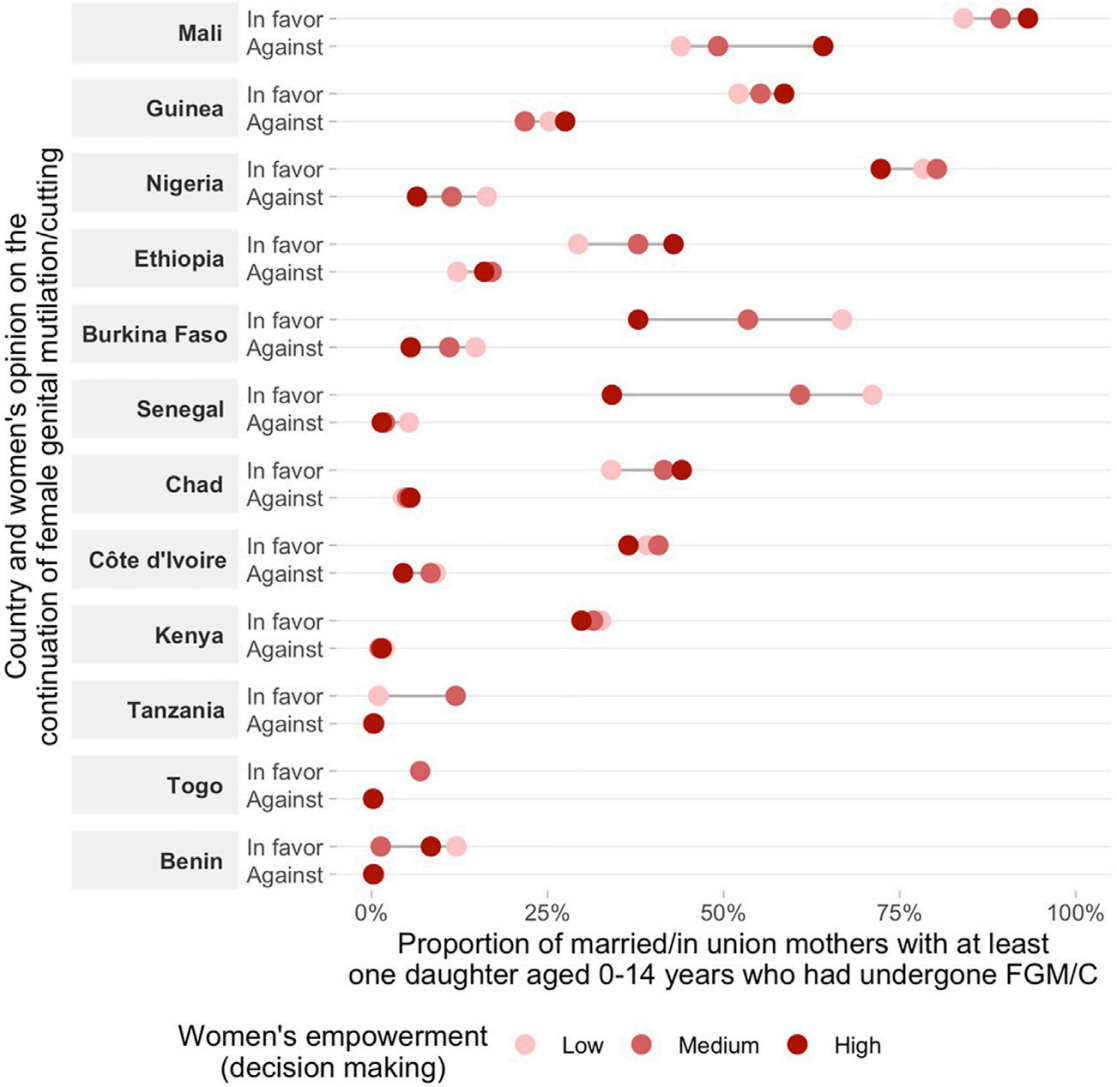
Double stratification of the daughters’ FGM/C according to the mothers’ attitudes towards FGM/C and women’s empowerment (decision making domain). Proportions are only presented for subgroups with at least 25 women. Results for subgroups with sample size smaller than 25 were suppressed from the figure.

**FIGURE 7:**
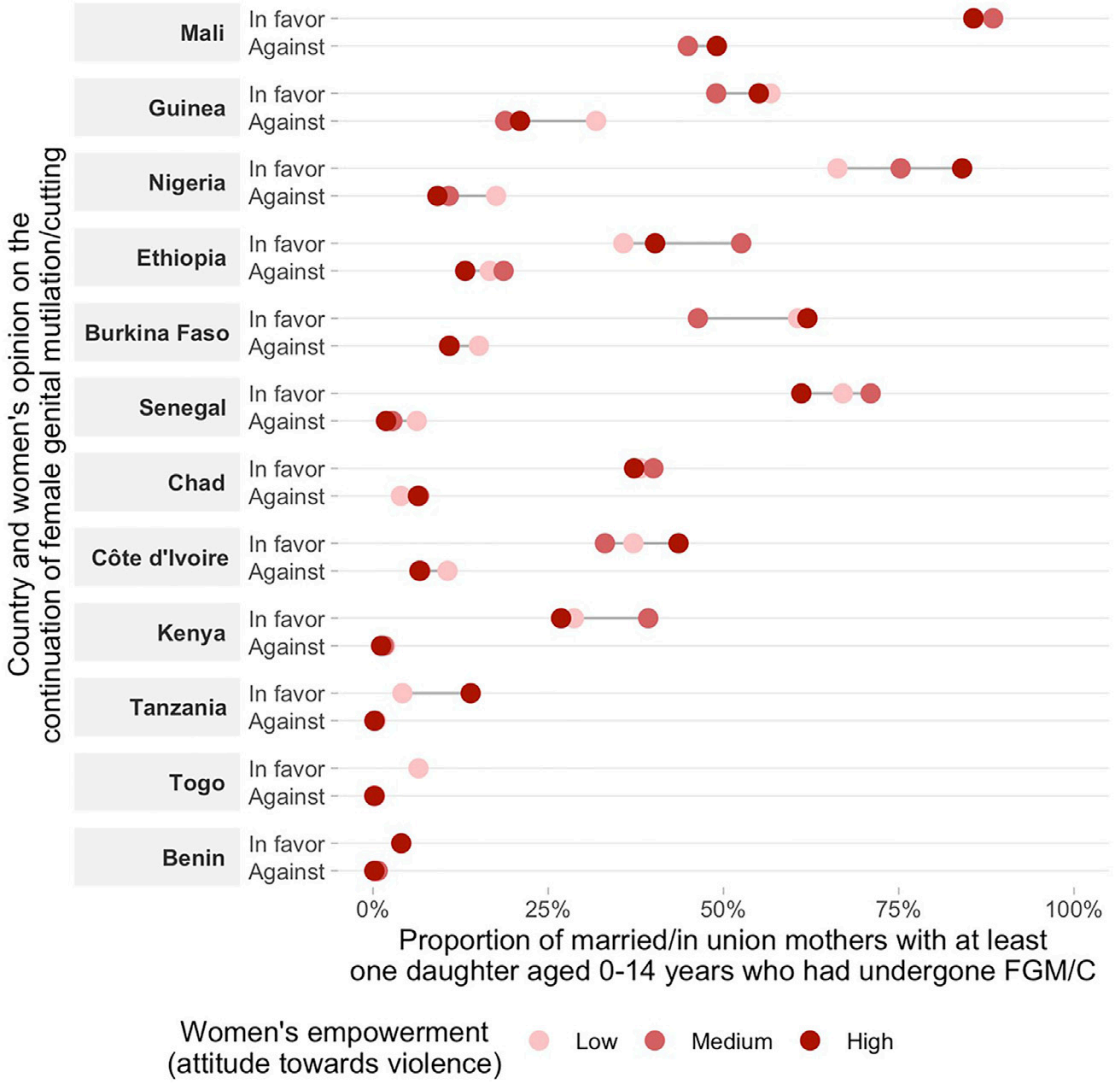
Double stratification of the daughters’ FGM/C according to the mothers’ attitudes towards FGM/C and women’s empowerment (attitude towards violence domain). Results for subgroups with sample size smaller than 25 were suppressed from the figure.

Double data disaggregation by women’s empowerment level (low, medium and high) and opinion towards the continuation of FGM/C allowed the examination of our outcome (proportion of women with at least one daughter aged 0–14 who had undergone FGM/C) in 6 groups of women. This allows the comparison of the outcome among the different categories of opinion towards FGM/C (in favor, and against) among women with similar levels of empowerment, although sample sizes vary in the 6 cells, with the “against” category having the smallest sample size. In accordance with our hypothesis, a lower proportion of daughters’ FGM/C was observed among women against the continuation of FGM/C and with a high empowerment level while a higher proportion of daughters’ FGM/C was observed among women in favor of FGM/C and with a low empowerment level in most countries (Figures 5–7 and Supplementary Tables S8–S10). This pattern was particularly evident for the social independence domain of empowerment (Figure 5).

In some countries, the proportion of daughters’ FGM/C varied significantly according to the women’s empowerment level, even among women who reported being in favor of the continuation of the practice. For instance, in Senegal, the proportion of women with at least one daughter who had undergone FGM/C was significantly lower among women with a high empowerment level as compared to those with low empowerment levels in the social independence and decision-making domains. For the decision-making domain the differences were quite substantial: 34.1% (95% CI: 25.9–43.4%) and 71.1% (95% CI: 67.4–74.6%), respectively. Other countries with marked differences were Nigeria, Ethiopia, Burkina-Faso and Côte d´Ivoire for the social independence domain. Nevertheless, the analyses showed markedly variable patterns by country. For the other two domains (decision-making and attitude to violence), no consistent patterns were found. In some countries, daughter’s FGM/C among women in favor of FGM/C was higher for women pertaining to the high empowerment group. This was the case for Mali, Chad, Guinea and Ethiopia (decision-making) and Nigeria, Côte d´Ivoire and Tanzania (attitude to violence).

## Discussion

Our findings point to the strong links between mother’s opinion towards the continuation of FGM/C, empowerment levels and daughter’s FGM/C. Overall, being against FGM/C and having a higher empowerment level were both individually related to lower levels of FGM/C among daughters. The investigation of the double stratification of the two characteristics revealed that the combination of having a favorable opinion towards FGM/C with women’s low empowerment levels was related to an increased risk of having at least one daughter undergoing FGM/C in many of the countries included in our analyses. Additionally, the double data disaggregation showed that a higher empowerment level was overall related to a lower national proportion of daughters’ FGM/C even among women in favor of the practice. All these findings were particularly consistent for the social independence domain of the women’s empowerment indicator.

In few countries, however, a higher empowerment level among women in favor of FGM/C continuation was related to a higher proportion of daughters’ FGM/C. This pattern was particularly noted for the decision-making domain of women’s empowerment and for those countries with higher prevalence of FGM/C (e.g., Mali, Guinea, and Ethiopia). Consistent with our findings, evidence from a study using DHS data from 15 African countries pointed that the opinion of the mother regarding the continuation of the practice exerts prominent influence in the decision making related to the cutting of girls (Hernlund and Shell-Duncan, 2007; Cappa et al., 2020). These results suggest that women’s decision power might exert an important role—to the point of prevailing over the husband’s/partner’s decision—in perpetuating the practice in societies in which FGM/C is still embedded in local tradition and culture and is widely supported at the community level. For instance, there is evidence that in Sierra Leone, contextual factors are responsible for more than half of the variability in the prevalence of FGM/C (Ameyaw et al., 2020). In traditional societies, the practice of FGM/C confers status on a woman because it promotes her the social identity and moral status within the community (e.g., parents fear that non-circumcised daughters will have more difficult to find a good marriage).

The extent to which individuals and families can withstand the pressure to conform the norms and go against its continuation is therefore dependent on their exposure to other social environments and influences. Anti-FGM/C attitudes are expected to increase over time with improvements in women’s social position leading to gradual shifts in societal attitudes (Van Rossem et al., 2015). Accordingly, our findings showed that a high empowerment level in the social independence domain which is composed of woman’s access to information, educational attainment, age at first marriage and first child, and difference in age and education between the woman and her husband was linked to a lower proportion of daughter’s FGM/C in most countries and, surprisingly, even for women in favor of the continuation of FGM/C. This could indicate that women with higher social independence might be exposed to a social environment that pressures against FGM/C, giving rise to hesitancy, delay or a shared decision making that helps to prevent the daughter’s FGM/C, even if the mother declares herself in favor of the practice.

In agreement with our hypothesis, an ethnographic study carried out in Guinea, which is among the countries with the highest prevalence of FGM/C worldwide, showed that among young women and men from different generations who do not practice FGM/C, having favorable social support and financial independence were key empowerment conditions allowing them to refuse to have their daughters cut (Doucet et al., 2020). The same study revealed the existence of distinct sociocultural profiles of people who do not practice FGM/C–varying from women who ended up being activists of FGM/C abandonment and openly speak about their decision to those who decided to lie about the FGM/C status of their daughters to avoid turmoil in a context of increased social pressure (Doucet et al., 2020).

Being against FGM/C in many countries may be extremely challenging for most women because FGM/C is a practice that gives them status and pride in societies (Agboli et al., 2020). Thus, many times, these women have insufficient knowledge of the several complications and risks of performing FGM/C (Agboli et al., 2020). A qualitative study in Belgium with immigrant women that have been subjected to FGM/C in their country of origin, showed that those who participated in FGM/C workshops not only increased their knowledge about health consequences of FGM/C but also became aware of FGM/C as a violation of human rights (Agboli et al., 2020). Besides, women living in this new context, with higher understanding about FGM/C and related social protection measures, were against the continuation of the practice in their daughters. Therefore, although women’s empowerment at the individual level is raised as a key factor for coping with FGM/C, there is a need for strategies to be extended to community/country level. In this sense, UNICEF and WHO recommends that approaches to reduce FGM/C prevalence at the country level combine women’s empowerment strategies with plans of action to build an environment that supports the change (e.g., by increasing community non-judgmental views) as key elements (UNICEF, 2005).

Countries where FGM/C is prevalent typically have equally higher overall levels of other types of gender-based violence and discrimination against women (Peltzer and Pengpid, 2014; Khosla et al., 2017). Interestingly, our findings showed that a higher empowerment level in the attitude to violence domain was overall related a lower proportion of women with at least one circumcised daughter. The overlap between multiple forms of gender-based violence is very common. A previous study from Kenya found that women who undergone FGM/C are more likely to justify intimate partner violence than those who have not (Sano et al., 2021). In Mali, women with FGM/C are at increased risk for intimate partner violence (Salihu et al., 2012). Strengthening policy links between the different forms of violence is recommended for improved sharing of knowledge and good practices around gender-based violence (Women U N, 2017).

On all fronts, the findings of our study reinforce the relevance of addressing social norms for change. The social norms theory has become a prominent approach for understanding the dynamics of behavior change, and in recent years has been increasingly adopted in the design of interventions aimed to promote collective community long-term abandonment of FGM/C. The importance of addressing community mobilization to reduce attitude towards FGM/C is already well recognized, and several interventions based on change in social norms of high gender inequality and violence against women and girls were already implemented in several countries (Ellsberg et al., 2015). The Saleema Initiative in South Sudan, for example, was effective in reducing pro-FGM/C social norms across 19 states by creating positive cultural associations with a girl remaining uncut. (Evans et al., 2019).

During the past 2 decades, great efforts have been made to eradicate FGM/C worldwide, including legal restrictions (WHO, 2020). The importance of the illegalization of the FGM/C goes behind the eradication of the practice itself. A potential favorable consequence of that policy is the change in social norms of strong gender inequalities such as the practice of bride price and lower education among women (García-Hombrados and Salgado, 2019). Among the countries included in our analysis, FGM/C is illegal in most of them since the late 1990s or early 2000s (Many Too, 2018). Exception are Mali and Chad, where there are still no legal restrictions to FGM/C (Many Too, 2018), and Nigeria, where the first law to prohibit FGM/C was implemented only in 2015 and it is not valid in all states (NPC, 2018; WHO, 2019). Our results highlight the low effectiveness of such legal institutions in several countries where prevalence of women with a young daughters who had undergone FGM/C is high. Another question is the possible under-reporting of FGM/C for girls born after the introduction of the law against the practice. Despite the confidentiality of the data, women may be afraid of confessing a crime (García-Hombrados and Salgado, 2019).

There are limitations to our analyses that should be considered. Firstly, the disclosure of both favorable opinion on the continuation of FGM/C and daughter’s FGM/C might be compromised due to increased advocacy on the elimination of the practice and ongoing legal restrictions. Therefore, we expect that our estimates for both outcomes might be underestimated (Bjälkander et al., 2013). Secondly, our sample includes women from a wide age range (15–49 years) and since older women tend to have more children (and therefore have a higher chance of having at least one daughter who had undergone FGM/C), the comparison of national FGM/C prevalence could be affected by different age structures between the studied countries. It should also be noted that information on the distinct types of FGM/C practice undergone by daughters–which can vary in terms of severity and associate short-term and long-term risks–was not assessed in the DHS, therefore limiting our ability to differentiate about the procedures made across countries. Thirdly, our empowerment indicator leaves out other important aspects of women’s agency and autonomy such as personal ownership of assets, economic participation, and opportunities and participation in governance processes which warrants future investigation. Forth, despite the recognition that men can play a role in the decision-making process regarding FGM/C, our study focused solely on the influence of mother-related factors which are known to exert a preponderant role in daughters FGM/C (Cappa et al., 2020). Finally, due to the cross-sectional nature of the study we could not stablish a causal relationship or temporal association between the variables studied. Hence, we could not affirm that a higher empowerment level has led to attitudes against FGM/C perpetuation which consequently led to the decision of not performing FGM/C among their daughters.

Despite the limitations, our study present innovative evidence on the intersectional effects of gender norms and social disadvantage in the prevalence of daughters’ FGM/C at the country level. Overall, a high empowerment level and being against FGM/C were linked to lower proportion of daughters’ undergoing FGM/C. But particularities observed such as the relationships variations by women’s empowerment domains and country level of FGM/C should be considered in intervention approaches. Strategies aimed for improvements in women’s agency and autonomy by increased education levels and social independence in addition to shifts in wider social norms supportive of FGM/C might contribute to pave the way for more significant reductions in FGM/C.

## Data Availability

The original contributions presented in the study are included in the article/Supplementary Material, further inquiries can be directed to the corresponding author.
