# Supplementary material for: Women’s Empowerment as It Relates to Attitudes Towards and Practice of Female Genital Mutilation/Cutting of Daughters: An Ecological Analysis of Demographic and Health Surveys From 12 African Countries
Source: Front Sociol. 2022 Jan 14;6:685329. doi: 10.3389/fsoc.2021.685329 (PMC8826721; doi:10.3389/fsoc.2021.685329)
Supplement: Supplementary file 1 [file DataSheet1.docx]

# Supplementary materials

Table S1. Description of included surveys.

| **Country** | **Year** | **Sample size** |
| --- | --- | --- |
| Benin | 2011 | 8,527 |
| Burkina Faso | 2010 | 9,454 |
| Chad | 2014 | 6,643 |
| Côte d’Ivoire | 2011 | 4,513 |
| Ethiopia | 2016 | 3,338 |
| Guinea | 2018 | 5,203 |
| Kenya | 2014 | 6,342 |
| Mali | 2018 | 2,942 |
| Nigeria | 2018 | 12,497 |
| Senegal | 2017 | 7,662 |
| Tanzania | 2015 | 5,599 |
| Togo | 2013 | 4,471 |

Table S2. Proportion of married/in union mothers with a favorable opinion on the continuation of female genital mutilation/cutting according to women’s empowerment (social independence)

| Country | Year | Women's empowerment (social independence) | n | Proportion of married/in union mothers with a favorable opinion on the continuation of FGM/C | Confidence interval (95%) |
| --- | --- | --- | --- | --- | --- |
| Mali | 2018 | Low | 1669 | 80.9% | (78.0 - 83.4) |
|  |  | Medium | 801 | 78.0% | (74.0 - 81.6) |
|  |  | High | 362 | 69.0% | (61.8 - 75.5) |
| Guinea | 2018 | Low | 3117 | 74.2% | (71.9 - 76.3) |
|  |  | Medium | 1326 | 69.1% | (65.7 - 72.3) |
|  |  | High | 679 | 65.2% | (60.1 - 70.0) |
| Nigeria | 2018 | Low | 6266 | 40.1% | (37.2 - 43.1) |
|  |  | Medium | 2847 | 22.9% | (19.7 - 26.5) |
|  |  | High | 3134 | 13.7% | (11.9 - 15.7) |
| Ethiopia | 2016 | Low | 2038 | 25.5% | (22.1 - 29.2) |
|  |  | Medium | 897 | 19.5% | (14.9 - 25.0) |
|  |  | High | 377 | 12.7% | (7.7 - 20.2) |
| Burkina Faso | 2010 | Low | 5872 | 10.6% | (9.2 - 12.3) |
|  |  | Medium | 2727 | 9.1% | (7.7 - 10.7) |
|  |  | High | 717 | 6.2% | (4.5 - 8.5) |
| Senegal | 2017 | Low | 3498 | 23.6% | (21.1 - 26.3) |
|  |  | Medium | 2148 | 18.0% | (15.6 - 20.7) |
|  |  | High | 1234 | 11.1% | (9.0 - 13.5) |
| Chad | 2014 | Low | 4471 | 36.7% | (33.2 - 40.5) |
|  |  | Medium | 1304 | 29.0% | (24.7 - 33.6) |
|  |  | High | 387 | 18.9% | (14.6 - 24.0) |
| Côte d'Ivoire | 2011 | Low | 2244 | 21.9% | (18.9 - 25.3) |
|  |  | Medium | 1313 | 16.5% | (13.4 - 20.2) |
|  |  | High | 525 | 8.7% | (5.8 - 13.0) |
| Kenya | 2014 | Low | 1828 | 15.6% | (13.7 - 17.8) |
|  |  | Medium | 2500 | 7.0% | (5.8 - 8.3) |
|  |  | High | 1882 | 2.8% | (2.2 - 3.5) |
| Tanzania | 2015 | Low | 1582 | 5.9% | (4.3 - 8.1) |
|  |  | Medium | 2476 | 3.9% | (2.7 - 5.5) |
|  |  | High | 1514 | 1.2% | (0.7 - 2.0) |
| Togo | 2013 | Low | 1875 | 2.0% | (1.2 - 3.4) |
|  |  | Medium | 1628 | 2.1% | (1.4 - 3.2) |
|  |  | High | 835 | 0.7% | (0.3 - 1.7) |
| Benin | 2011 | Low | 4109 | 2.7% | (2.0 - 3.6) |
|  |  | Medium | 2583 | 2.5% | (1.8 - 3.6) |
|  |  | High | 1370 | 2.0% | (1.1 - 3.5) |

Table S3. Proportion of married/in union mothers with a favorable opinion on the continuation of female genital mutilation/cutting according to women’s empowerment (decision making)

| Country | Year | Women's empowerment (decision making) | n | Proportion of married/in union mothers with a favorable opinion on the continuation of FGM/C | Confidence interval (95%) |
| --- | --- | --- | --- | --- | --- |
| Mali | 2018 | Low | 1817 | 78.6% | (75.5 - 81.4) |
|  |  | Medium | 701 | 80.7% | (76.7 - 84.1) |
|  |  | High | 314 | 74.1% | (66.8 - 80.3) |
| Guinea | 2018 | Low | 1952 | 74.0% | (70.5 - 77.2) |
|  |  | Medium | 1704 | 70.4% | (67.4 - 73.3) |
|  |  | High | 1466 | 70.2% | (66.5 - 73.7) |
| Nigeria | 2018 | Low | 4831 | 39.1% | (36.1 - 42.2) |
|  |  | Medium | 3536 | 31.3% | (28.3 - 34.4) |
|  |  | High | 3880 | 15.4% | (13.4 - 17.7) |
| Ethiopia | 2016 | Low | 360 | 27.8% | (20.6 - 36.4) |
|  |  | Medium | 688 | 24.7% | (19.8 - 30.3) |
|  |  | High | 2264 | 21.2% | (18.0 - 24.8) |
| Burkina Faso | 2010 | Low | 3916 | 10.6% | (8.9 - 12.6) |
|  |  | Medium | 4354 | 9.8% | (8.4 - 11.5) |
|  |  | High | 1046 | 7.2% | (5.5 - 9.5) |
| Senegal | 2017 | Low | 4390 | 24.7% | (22.0 - 27.5) |
|  |  | Medium | 1655 | 9.8% | (8.1 - 11.7) |
|  |  | High | 835 | 14.3% | (11.2 - 18.0) |
| Chad | 2014 | Low | 2621 | 41.1% | (36.6 - 45.7) |
|  |  | Medium | 2574 | 29.8% | (25.9 - 34.1) |
|  |  | High | 967 | 27.0% | (22.9 - 31.5) |
| Côte d'Ivoire | 2011 | Low | 1755 | 21.1% | (17.2 - 25.5) |
|  |  | Medium | 1453 | 14.1% | (12.0 - 16.5) |
|  |  | High | 874 | 18.8% | (13.2 - 26.2) |
| Kenya | 2014 | Low | 698 | 14.5% | (11.7 - 17.9) |
|  |  | Medium | 2151 | 6.3% | (5.3 - 7.4) |
|  |  | High | 3361 | 7.2% | (6.1 - 8.5) |
| Tanzania | 2015 | Low | 981 | 9.3% | (6.3 - 13.3) |
|  |  | Medium | 2693 | 3.3% | (2.5 - 4.6) |
|  |  | High | 1898 | 1.3% | (0.8 - 2.0) |
| Togo | 2013 | Low | 1061 | 2.0% | (1.2 - 3.2) |
|  |  | Medium | 2071 | 1.6% | (1.0 - 2.6) |
|  |  | High | 1206 | 1.6% | (0.8 - 3.0) |
| Benin | 2011 | Low | 1857 | 2.7% | (1.8 - 4.2) |
|  |  | Medium | 2493 | 3.9% | (2.8 - 5.4) |
|  |  | High | 3712 | 1.3% | (0.9 - 2.0) |

Table S4. Proportion of married/in union mothers with a favorable opinion on the continuation of female genital mutilation/cutting according to women’s empowerment (attitude towards violence)

| Country | Year | Women's empowerment (attitude towards violence) | n | Proportion of married/in union mothers with a favorable opinion on the continuation of FGM/C | Confidence interval (95%) |
| --- | --- | --- | --- | --- | --- |
| Mali | 2018 | Low | 1609 | 81.6% | (78.7 - 84.1%) |
|  |  | Medium | 637 | 73.9% | (69.3 - 78.0%) |
|  |  | High | 586 | 74.9% | (69.6 - 79.5%) |
| Guinea | 2018 | Low | 2863 | 78.4% | (76.0 - 80.6%) |
|  |  | Medium | 887 | 63.4% | (59.3 - 67.3%) |
|  |  | High | 1372 | 63.4% | (59.1 - 67.6%) |
| Nigeria | 2018 | Low | 2805 | 42.9% | (39.4 - 46.6%) |
|  |  | Medium | 1216 | 32.8% | (28.2 - 37.8%) |
|  |  | High | 8226 | 23.9% | (21.7 - 26.2%) |
| Ethiopia | 2016 | Low | 1458 | 24.9% | (21.1 - 29.2%) |
|  |  | Medium | 610 | 23.3% | (18.3 - 29.2%) |
|  |  | High | 1244 | 18.4% | (15.0 - 22.5%) |
| Burkina Faso | 2010 | Low | 2357 | 15.4% | (13.0 - 18.1%) |
|  |  | Medium | 1980 | 10.2% | (8.5 - 12.3%) |
|  |  | High | 4979 | 7.0% | (5.9 - 8.3%) |
| Senegal | 2017 | Low | 3391 | 25.2% | (22.5 - 28.1%) |
|  |  | Medium | 770 | 16.4% | (13.5 - 19.9%) |
|  |  | High | 2719 | 13.9% | (12.0 - 16.1%) |
| Chad | 2014 | Low | 3317 | 31.0% | (27.4 - 34.9%) |
|  |  | Medium | 1345 | 28.8% | (24.5 - 33.5%) |
|  |  | High | 1500 | 46.4% | (39.7 - 53.1%) |
| Côte d'Ivoire | 2011 | Low | 1332 | 23.8% | (18.9 - 29.5%) |
|  |  | Medium | 809 | 19.0% | (15.2 - 23.4%) |
|  |  | High | 1941 | 14.6% | (11.8 - 17.9%) |
| Kenya | 2014 | Low | 1509 | 12.2% | (10.3 - 14.4%) |
|  |  | Medium | 1700 | 8.1% | (6.7 - 9.7%) |
|  |  | High | 3001 | 5.6% | (4.8 - 6.6%) |
| Tanzania | 2015 | Low | 2206 | 5.5% | (3.9 - 7.7%) |
|  |  | Medium | 1063 | 2.2% | (1.4 - 3.5%) |
|  |  | High | 2303 | 2.3% | (1.6 - 3.2%) |
| Togo | 2013 | Low | 926 | 4.0% | (2.5 - 6.5%) |
|  |  | Medium | 710 | 1.9% | (0.9 - 3.9%) |
|  |  | High | 2702 | 1.0% | (0.6 - 1.6%) |
| Benin | 2011 | Low | 706 | 4.1% | (2.5 - 6.7%) |
|  |  | Medium | 890 | 1.9% | (0.9 - 3.6%) |
|  |  | High | 6466 | 2.4% | (1.8 - 3.2%) |

Table S5. Proportion of married/in union mothers with at least one daughter aged 0-14 years who had undergone female genital mutilation/cutting according to women’s empowerment (social independence)

| Country | Year | Women's empowerment (social independence) | n | Proportion of married/in union mothers with at least one daughter who had undergone FGM/C | Confidence interval (95%) |
| --- | --- | --- | --- | --- | --- |
| Mali | 2018 | Low | 1669 | 77.5% | (74.8 - 80.0%) |
|  |  | Medium | 801 | 75.2% | (71.4 - 78.7%) |
|  |  | High | 362 | 70.7% | (65.0 - 75.9%) |
| Guinea | 2018 | Low | 3117 | 50.3% | (47.9 - 52.8%) |
|  |  | Medium | 1326 | 44.5% | (41.5 - 47.6%) |
|  |  | High | 679 | 40.0% | (35.6 - 44.5%) |
| Nigeria | 2018 | Low | 6266 | 25.8% | (23.5 - 28.1%) |
|  |  | Medium | 2847 | 16.5% | (14.4 - 19.0%) |
|  |  | High | 3134 | 11.1% | (9.6 - 12.7%) |
| Ethiopia | 2016 | Low | 2038 | 23.1% | (19.8 - 26.7%) |
|  |  | Medium | 897 | 15.3% | (12.1 - 19.1%) |
|  |  | High | 377 | 9.8% | (6.4 - 14.5%) |
| Burkina Faso | 2010 | Low | 5872 | 18.1% | (16.4 - 19.9%) |
|  |  | Medium | 2727 | 14.9% | (13.2 - 16.8%) |
|  |  | High | 717 | 10.2% | (8.0 - 13.0%) |
| Senegal | 2017 | Low | 3498 | 19.7% | (17.3 - 22.3%) |
|  |  | Medium | 2148 | 13.4% | (11.5 - 15.5%) |
|  |  | High | 1234 | 7.0% | (5.4 - 9.0%) |
| Chad | 2014 | Low | 4471 | 14.6% | (13.0 - 16.4%) |
|  |  | Medium | 1304 | 11.2% | (9.1 - 13.7%) |
|  |  | High | 387 | 8.7% | (5.8 - 12.9%) |
| Côte d'Ivoire | 2011 | Low | 2244 | 15.3% | (13.1 - 17.7%) |
|  |  | Medium | 1313 | 12.5% | (10.2 - 15.2%) |
|  |  | High | 525 | 4.3% | (2.5 - 7.1%) |
| Kenya | 2014 | Low | 1828 | 6.2% | (5.1 - 7.5%) |
|  |  | Medium | 2500 | 3.3% | (2.6 - 4.3%) |
|  |  | High | 1882 | 1.8% | (1.3 - 2.5%) |
| Tanzania | 2015 | Low | 1582 | 0.5% | (0.2 - 1.1%) |
|  |  | Medium | 2476 | 0.5% | (0.3 - 0.8%) |
|  |  | High | 1514 | 0.2% | (0.1 - 0.6%) |
| Togo | 2013 | Low | 1875 | 0.3% | (0.1 - 0.7%) |
|  |  | Medium | 1628 | 0.5% | (0.3 - 1.0%) |
|  |  | High | 835 | 0.1% | (0.0 - 0.6%) |
| Benin | 2011 | Low | 4109 | 0.3% | (0.2 - 0.6%) |
|  |  | Medium | 2583 | 0.1% | (0.1 - 0.3%) |
|  |  | High | 1370 | 0.2% | (0.1 - 0.6%) |

Table S6. Proportion of married/in union mothers with at least one daughter aged 0-14 years who had undergone female genital mutilation/cutting according to women’s empowerment (decision making)

| Country | Year | Women's empowerment (decision making) | n | Proportion of married/in union mothers with at least one daughter who had undergone FGM/C | Confidence interval (95%) |
| --- | --- | --- | --- | --- | --- |
| Mali | 2018 | Low | 1817 | 73.1% | (70.1 - 75.9%) |
|  |  | Medium | 701 | 81.2% | (77.5 - 84.4%) |
|  |  | High | 314 | 81.7% | (75.1 - 86.9%) |
| Guinea | 2018 | Low | 1952 | 45.5% | (42.8 - 48.2%) |
|  |  | Medium | 1704 | 47.4% | (44.6 - 50.2%) |
|  |  | High | 1466 | 50.0% | (46.6 - 53.5%) |
| Nigeria | 2018 | Low | 4831 | 25.0% | (22.6 - 27.5%) |
|  |  | Medium | 3536 | 20.3% | (18.2 - 22.5%) |
|  |  | High | 3880 | 13.2% | (11.3 - 15.4%) |
| Ethiopia | 2016 | Low | 360 | 14.5% | (10.4 - 19.8%) |
|  |  | Medium | 688 | 20.4% | (16.0 - 25.5%) |
|  |  | High | 2264 | 20.3% | (17.2 - 23.7%) |
| Burkina Faso | 2010 | Low | 3916 | 20.5% | (18.2 - 23.0%) |
|  |  | Medium | 4354 | 15.2% | (13.6 - 17.1%) |
|  |  | High | 1046 | 8.1% | (6.3 - 10.2%) |
| Senegal | 2017 | Low | 4390 | 20.6% | (18.1 - 23.4%) |
|  |  | Medium | 1655 | 7.6% | (6.1 - 9.4%) |
|  |  | High | 835 | 6.0% | (4.5 - 7.8%) |
| Chad | 2014 | Low | 2621 | 13.4% | (11.6 - 15.4%) |
|  |  | Medium | 2574 | 13.2% | (11.2 - 15.6%) |
|  |  | High | 967 | 14.5% | (11.9 - 17.5%) |
| Côte d'Ivoire | 2011 | Low | 1755 | 14.6% | (11.9 - 17.6%) |
|  |  | Medium | 1453 | 12.5% | (10.3 - 15.0%) |
|  |  | High | 874 | 10.0% | (7.3 - 13.5%) |
| Kenya | 2014 | Low | 698 | 5.9% | (4.2 - 8.1%) |
|  |  | Medium | 2151 | 2.9% | (2.3 - 3.6%) |
|  |  | High | 3361 | 3.5% | (2.8 - 4.2%) |
| Tanzania | 2015 | Low | 981 | 0.1% | (0.0 - 0.5%) |
|  |  | Medium | 2693 | 0.6% | (0.4 - 1.1%) |
|  |  | High | 1898 | 0.3% | (0.1 - 0.7%) |
| Togo | 2013 | Low | 1061 | 0.5% | (0.3 - 1.1%) |
|  |  | Medium | 2071 | 0.3% | (0.1 - 0.6%) |
|  |  | High | 1206 | 0.3% | (0.1 - 0.9%) |
| Benin | 2011 | Low | 1857 | 0.4% | (0.1 - 0.9%) |
|  |  | Medium | 2493 | 0.3% | (0.1 - 0.6%) |
|  |  | High | 3712 | 0.2% | (0.1 - 0.4%) |

Table S7. Proportion of married/in union mothers with at least one daughter aged 0-14 years who had undergone female genital mutilation/cutting according to women’s empowerment (attitude towards violence)

| Country | Year | Women's empowerment (attitude towards violence) | n | Proportion of married/in union mothers with at least one daughter who had undergone FGM/C | Confidence interval (95%) |
| --- | --- | --- | --- | --- | --- |
| Mali | 2018 | Low | 1609 | 77.6% | (74.9 - 80.1%) |
|  |  | Medium | 637 | 76.0% | (71.8 - 79.8%) |
|  |  | High | 586 | 70.7% | (65.6 - 75.2%) |
| Guinea | 2018 | Low | 2863 | 51.7% | (49.1 - 54.3%) |
|  |  | Medium | 887 | 40.6% | (36.3 - 45.0%) |
|  |  | High | 1372 | 43.5% | (40.1 - 47.0%) |
| Nigeria | 2018 | Low | 2805 | 21.1% | (18.6 - 23.8%) |
|  |  | Medium | 1216 | 19.6% | (16.6 - 23.0%) |
|  |  | High | 8226 | 19.5% | (17.7 - 21.5%) |
| Ethiopia | 2016 | Low | 1458 | 20.2% | (17.1 - 23.6%) |
|  |  | Medium | 610 | 24.3% | (19.5 - 29.9%) |
|  |  | High | 1244 | 16.3% | (13.0 - 20.2%) |
| Burkina Faso | 2010 | Low | 2357 | 22.3% | (19.6 - 25.3%) |
|  |  | Medium | 1980 | 14.6% | (12.7 - 16.7%) |
|  |  | High | 4979 | 14.5% | (13.1 - 16.1%) |
| Senegal | 2017 | Low | 3391 | 20.5% | (17.9 - 23.5%) |
|  |  | Medium | 770 | 13.2% | (10.7 - 16.1%) |
|  |  | High | 2719 | 10.0% | (8.4 - 11.9%) |
| Chad | 2014 | Low | 3317 | 11.7% | (10.1 - 13.5%) |
|  |  | Medium | 1345 | 14.1% | (11.5 - 17.3%) |
|  |  | High | 1500 | 17.9% | (14.8 - 21.5%) |
| Côte d'Ivoire | 2011 | Low | 1332 | 16.5% | (13.5 - 19.9%) |
|  |  | Medium | 809 | 10.9% | (8.2 - 14.2%) |
|  |  | High | 1941 | 11.4% | (9.2 - 14.0%) |
| Kenya | 2014 | Low | 1509 | 4.8% | (3.7 - 6.1%) |
|  |  | Medium | 1700 | 4.4% | (3.4 - 5.6%) |
|  |  | High | 3001 | 2.6% | (2.1 - 3.3%) |
| Tanzania | 2015 | Low | 2206 | 0.5% | (0.3 - 1.0%) |
|  |  | Medium | 1063 | 0.2% | (0.1 - 0.8%) |
|  |  | High | 2303 | 0.4% | (0.2 - 0.8%) |
| Togo | 2013 | Low | 926 | 0.4% | (0.2 - 1.0%) |
|  |  | Medium | 710 | 0.3% | (0.1 - 1.0%) |
|  |  | High | 2702 | 0.3% | (0.2 - 0.6%) |
| Benin | 2011 | Low | 706 | 0.6% | (0.2 - 1.7%) |
|  |  | Medium | 890 | 0.6% | (0.2 - 1.7%) |
|  |  | High | 6466 | 0.2% | (0.1 - 0.3%) |

Table S8. Double stratification of the daughters’ FGM/C according to attitudes towards female genital mutilation/cutting and women’s empowerment (social independence)

| Country | Year | Women's empowerment (social independence) | Women's opinion on the continuation of FGM/C | n | Proportion of married/in union mothers with at least one daughter who had undergone FGM/C | Confidence interval (95%) |
| --- | --- | --- | --- | --- | --- | --- |
| Mali | 2018 | Low | In favor | 1160 | 87.1% | (84.5 - 89.4%) |
|  |  |  | Depends | 42 | 70.6% | (51.9 - 84.2%) |
|  |  |  | Against | 282 | 49.4% | (40.9 - 57.9%) |
|  |  |  | Don't know | 28 | 64.0% | (42.2 - 81.3%) |
|  |  | Medium | In favor | 563 | 84.7% | (81.2 - 87.6%) |
|  |  |  | Depends | 31 | 68.8% | (50.1 - 82.9%) |
|  |  |  | Against | 136 | 47.7% | (36.2 - 59.4%) |
|  |  |  | Don't know | 8 | -^1^ | -^1^ |
|  |  | High | In favor | 203 | 86.5% | (81.0 - 90.5%) |
|  |  |  | Depends | 13 | -^1^ | -^1^ |
|  |  |  | Against | 90 | 43.8% | (31.5 - 56.9%) |
|  |  |  | Don't know | 8 | -^1^ | -^1^ |
| Guinea | 2018 | Low | In favor | 2290 | 57.0% | (54.2 - 59.8%) |
|  |  |  | Depends | 118 | 54.7% | (45.2 - 64.0%) |
|  |  |  | Against | 595 | 27.2% | (23.2 - 31.7%) |
|  |  |  | Don't know | 80 | 41.6% | (29.4 - 55.0%) |
|  |  | Medium | In favor | 899 | 53.0% | (49.4 - 56.5%) |
|  |  |  | Depends | 64 | 42.3% | (31.0 - 54.4%) |
|  |  |  | Against | 296 | 24.4% | (19.7 - 29.8%) |
|  |  |  | Don't know | 43 | 33.3% | (20.0 - 49.8%) |
|  |  | High | In favor | 456 | 48.7% | (43.2 - 54.2%) |
|  |  |  | Depends | 29 | 59.5% | (38.1 - 77.8%) |
|  |  |  | Against | 169 | 18.6% | (12.7 - 26.5%) |
|  |  |  | Don't know | 16 | -^1^ | -^1^ |
| Nigeria | 2018 | Low | In favor | 1335 | 80.3% | (77.2 - 83.2%) |
|  |  |  | Depends | 384 | 33.7% | (27.3 - 40.6%) |
|  |  |  | Against | 1904 | 17.1% | (15.0 - 19.5%) |
|  |  |  | Don't know | 24 | -^1^ | -^1^ |
|  |  | Medium | In favor | 374 | 77.4% | (71.8 - 82.2%) |
|  |  |  | Depends | 131 | 37.5% | (28.6 - 47.3%) |
|  |  |  | Against | 1181 | 7.5% | (5.8 - 9.6%) |
|  |  |  | Don't know | 24 | -^1^ | -^1^ |
|  |  | High | In favor | 299 | 65.7% | (58.2 - 72.5%) |
|  |  |  | Depends | 89 | 25.4% | (15.2 - 39.3%) |
|  |  |  | Against | 1747 | 6.3% | (5.1 - 7.8%) |
|  |  |  | Don't know | 25 | 23.2% | (9.6 - 46.1%) |
| Ethiopia | 2016 | Low | In favor | 598 | 43.8% | (37.1 - 50.9%) |
|  |  |  | Depends | 32 | 23.1% | (11.8 - 40.2%) |
|  |  |  | Against | 1163 | 18.9% | (15.5 - 22.8%) |
|  |  |  | Don't know | 39 | 24.9% | (13.3 - 41.7%) |
|  |  | Medium | In favor | 177 | 33.6% | (25.7 - 42.5%) |
|  |  |  | Depends | 7 | -^1^ | -^1^ |
|  |  |  | Against | 589 | 12.2% | (9.2 - 16.0%) |
|  |  |  | Don't know | 13 | -^1^ | -^1^ |
|  |  | High | In favor | 48 | 19.5% | (11.4 - 31.4%) |
|  |  |  | Depends | 2 | -^1^ | -^1^ |
|  |  |  | Against | 307 | 9.2% | (5.5 - 14.9%) |
|  |  |  | Don't know | 3 | -^1^ | -^1^ |
| Burkina Faso | 2010 | Low | In favor | 555 | 59.8% | (54.5 - 64.9%) |
|  |  |  | Depends | 42 | 43.1% | (25.8 - 62.3%) |
|  |  |  | Against | 5237 | 13.0% | (11.7 - 14.4%) |
|  |  |  | Don't know | 8 | -^1^ | -^1^ |
|  |  | Medium | In favor | 227 | 54.7% | (48.0 - 61.3%) |
|  |  |  | Depends | 19 | -^1^ | -^1^ |
|  |  |  | Against | 2472 | 10.8% | (9.3 - 12.4%) |
|  |  |  | Don't know | 4 | -^1^ | -^1^ |
|  |  | High | In favor | 39 | 51.4% | (32.3 - 70.1%) |
|  |  |  | Depends | 4 | -^1^ | -^1^ |
|  |  |  | Against | 672 | 7.4% | (5.5 - 10.0%) |
|  |  |  | Don't know | 1 | -^1^ | -^1^ |
| Senegal | 2017 | Low | In favor | 994 | 70.9% | (66.8 - 74.6%) |
|  |  |  | Depends | 73 | 11.9% | (6.0 - 22.5%) |
|  |  |  | Against | 2073 | 5.2% | (4.3 - 6.3%) |
|  |  |  | Don't know | 103 | 11.8% | (6.2 - 21.4%) |
|  |  | Medium | In favor | 492 | 63.2% | (58.1 - 68.1%) |
|  |  |  | Depends | 31 | 10.2% | (2.5 - 33.9%) |
|  |  |  | Against | 1450 | 2.8% | (2.2 - 3.6%) |
|  |  |  | Don't know | 55 | 14.6% | (7.8 - 25.6%) |
|  |  | High | In favor | 181 | 47.5% | (38.4 - 56.7%) |
|  |  |  | Depends | 11 | -^1^ | -^1^ |
|  |  |  | Against | 994 | 2.0% | (1.3 - 3.1%) |
|  |  |  | Don't know | 15 | -^1^ | -^1^ |
| Chad | 2014 | Low | In favor | 1387 | 38.5% | (34.6 - 42.6%) |
|  |  |  | Depends | 434 | 13.0% | (9.8 - 17.1%) |
|  |  |  | Against | 1241 | 5.6% | (4.2 - 7.4%) |
|  |  |  | Don't know | 399 | 5.9% | (3.9 - 9.0%) |
|  |  | Medium | In favor | 316 | 37.2% | (30.8 - 44.1%) |
|  |  |  | Depends | 159 | 11.6% | (6.1 - 20.8%) |
|  |  |  | Against | 408 | 3.1% | (1.7 - 5.9%) |
|  |  |  | Don't know | 110 | 3.9% | (1.2 - 11.8%) |
|  |  | High | In favor | 71 | 37.0% | (24.7 - 51.1%) |
|  |  |  | Depends | 47 | 2.1% | (0.3 - 12.2%) |
|  |  |  | Against | 165 | 4.8% | (1.6 - 13.8%) |
|  |  |  | Don't know | 30 | 4.9% | (0.4 - 38.7%) |
| Côte d'Ivoire | 2011 | Low | In favor | 461 | 40.3% | (34.1 - 46.9%) |
|  |  |  | Depends | 39 | 19.4% | (9.2 - 36.5%) |
|  |  |  | Against | 1421 | 9.7% | (7.8 - 12.0%) |
|  |  |  | Don't know | 55 | 18.2% | (8.1 - 35.8%) |
|  |  | Medium | In favor | 201 | 37.9% | (29.6 - 46.9%) |
|  |  |  | Depends | 20 | -^1^ | -^1^ |
|  |  |  | Against | 895 | 7.8% | (5.6 - 10.6%) |
|  |  |  | Don't know | 32 | 9.6% | (3.3 - 25.0%) |
|  |  | High | In favor | 37 | 30.4% | (12.6 - 57.0%) |
|  |  |  | Depends | 8 | -^1^ | -^1^ |
|  |  |  | Against | 441 | 2.0% | (1.2 - 3.3%) |
|  |  |  | Don't know | 4 | -^1^ | -^1^ |
| Kenya | 2014 | Low | In favor | 493 | 32.7% | (27.1 - 38.9%) |
|  |  |  | Depends | 30 | 5.2% | (1.0 - 22.9%) |
|  |  |  | Against | 1204 | 1.8% | (1.3 - 2.6%) |
|  |  |  | Don't know | 8 | -^1^ | -^1^ |
|  |  | Medium | In favor | 303 | 28.4% | (19.9 - 38.9%) |
|  |  |  | Depends | 30 | 10.3% | (2.7 - 31.7%) |
|  |  |  | Against | 2064 | 1.5% | (1.0 - 2.1%) |
|  |  |  | Don't know | 10 | -^1^ | -^1^ |
|  |  | High | In favor | 106 | 30.5% | (21.1 - 41.8%) |
|  |  |  | Depends | 17 | -^1^ | -^1^ |
|  |  |  | Against | 1726 | 1.0% | (0.6 - 1.7%) |
|  |  |  | Don't know | 3 | -^1^ | -^1^ |
| Tanzania | 2015 | Low | In favor | 58 | 7.5% | (2.6 - 19.7%) |
|  |  |  | Depends | 12 | -^1^ | -^1^ |
|  |  |  | Against | 988 | 0.2% | (0.1 - 0.9%) |
|  |  |  | Don't know | 15 | -^1^ | -^1^ |
|  |  | Medium | In favor | 68 | 5.2% | (2.4 - 11.1%) |
|  |  |  | Depends | 18 | -^1^ | -^1^ |
|  |  |  | Against | 1831 | 0.5% | (0.2 - 0.8%) |
|  |  |  | Don't know | 36 | 0.0% | -^2^ |
|  |  | High | In favor | 18 | -^1^ | -^1^ |
|  |  |  | Depends | 9 | -^1^ | -^1^ |
|  |  |  | Against | 1274 | 0.2% | (0.0 - 0.6%) |
|  |  |  | Don't know | 9 | -^1^ | -^1^ |
| Togo | 2013 | Low | In favor | 25 | 2.9% | (0.3 - 24.3%) |
|  |  |  | Depends | 26 | 4.2% | (0.8 - 18.9%) |
|  |  |  | Against | 1208 | 0.4% | (0.1 - 1.1%) |
|  |  |  | Don't know | 9 | -^1^ | -^1^ |
|  |  | Medium | In favor | 25 | 20.8% | (7.0 - 47.7%) |
|  |  |  | Depends | 30 | 4.2% | (0.9 - 17.2%) |
|  |  |  | Against | 1048 | 0.2% | (0.1 - 0.6%) |
|  |  |  | Don't know | 13 | -^1^ | -^1^ |
|  |  | High | In favor | 5 | -^1^ | -^1^ |
|  |  |  | Depends | 16 | -^1^ | -^1^ |
|  |  |  | Against | 608 | 0.0% | -^2^ |
|  |  |  | Don't know | 8 | -^1^ | -^1^ |
| Benin | 2011 | Low | In favor | 76 | 7.5% | (2.4 - 21.2%) |
|  |  |  | Depends | 148 | 0.0% | -^2^ |
|  |  |  | Against | 2421 | 0.3% | (0.2 - 0.7%) |
|  |  |  | Don't know | 58 | 1.3% | (0.1 - 11.0%) |
|  |  | Medium | In favor | 46 | 1.8% | (0.2 - 15.3%) |
|  |  |  | Depends | 81 | 0.0% | -^2^ |
|  |  |  | Against | 1536 | 0.2% | (0.1 - 0.5%) |
|  |  |  | Don't know | 28 | 0.0% | - |
|  |  | High | In favor | 20 | -^1^ | -^1^ |
|  |  |  | Depends | 27 | 0.0% | -^2^ |
|  |  |  | Against | 884 | 0.2% | (0.0 - 0.8%) |
|  |  |  | Don't know | 17 | -^1^ | -^1^ |

^1^ The proportion was not calculated for subgroups with less than 25 women

^2^ The confidence interval was not calculated if there were no women with at least one daughter who had undergone FGM/C in the subgroup

Table S9. Double stratification of the daughters’ FGM/C according to attitudes towards female genital mutilation/cutting and women’s empowerment (decision making)

| Country | Year | Women's empowerment (decision making) | Women's opinion on the continuation of FGM/C | n | Proportion of married/in union mothers with at least one daughter who had undergone FGM/C | Confidence interval (95%) |
| --- | --- | --- | --- | --- | --- | --- |
| Mali | 2018 | Low | In favor | 1245 | 84.1% | (80.9 - 86.7%) |
|  |  |  | Depends | 55 | 67.1% | (51.9 - 79.5%) |
|  |  |  | Against | 303 | 43.9% | (36.3 - 51.8%) |
|  |  |  | Don't know | 36 | 74.1% | (56.2 - 86.4%) |
|  |  | Medium | In favor | 503 | 89.3% | (86.3 - 91.8%) |
|  |  |  | Depends | 22 | -^1^ | -^1^ |
|  |  |  | Against | 119 | 49.2% | (38.0 - 60.5%) |
|  |  |  | Don't know | 6 | -^1^ | -^1^ |
|  |  | High | In favor | 178 | 93.2% | (88.2 - 96.2%) |
|  |  |  | Depends | 9 | -^1^ | -^1^ |
|  |  |  | Against | 86 | 64.1% | (51.0 - 75.4%) |
|  |  |  | Don't know | 2 | -^1^ | -^1^ |
| Guinea | 2018 | Low | In favor | 1434 | 52.1% | (48.9 - 55.3%) |
|  |  |  | Depends | 60 | 48.6% | (36.7 - 60.6%) |
|  |  |  | Against | 358 | 25.3% | (20.7 - 30.4%) |
|  |  |  | Don't know | 64 | 36.0% | (18.7 - 57.9%) |
|  |  | Medium | In favor | 1198 | 55.2% | (51.9 - 58.5%) |
|  |  |  | Depends | 99 | 54.9% | (44.8 - 64.6%) |
|  |  |  | Against | 327 | 21.7% | (17.4 - 26.9%) |
|  |  |  | Don't know | 57 | 35.4% | (22.0 - 51.5%) |
|  |  | High | In favor | 1013 | 58.6% | (54.5 - 62.5%) |
|  |  |  | Depends | 52 | 48.7% | (35.0 - 62.5%) |
|  |  |  | Against | 375 | 27.5% | (22.1 - 33.7%) |
|  |  |  | Don't know | 18 | -^1^ | -^1^ |
| Nigeria | 2018 | Low | In favor | 993 | 78.3% | (74.5 - 81.7%) |
|  |  |  | Depends | 255 | 39.9% | (31.8 - 48.5%) |
|  |  |  | Against | 1409 | 16.4% | (14.0 - 19.0%) |
|  |  |  | Don't know | 16 | -^1^ | -^1^ |
|  |  | Medium | In favor | 624 | 80.3% | (76.3 - 83.7%) |
|  |  |  | Depends | 187 | 28.1% | (20.9 - 36.6%) |
|  |  |  | Against | 1336 | 11.4% | (9.5 - 13.5%) |
|  |  |  | Don't know | 22 | -^1^ | -^1^ |
|  |  | High | In favor | 391 | 72.3% | (66.2 - 77.7%) |
|  |  |  | Depends | 162 | 29.5% | (22.9 - 36.9%) |
|  |  |  | Against | 2087 | 6.4% | (5.2 - 8.0%) |
|  |  |  | Don't know | 35 | 15.5% | (6.5 - 32.5%) |
| Ethiopia | 2016 | Low | In favor | 107 | 29.3% | (18.3 - 43.5%) |
|  |  |  | Depends | 5 | -^1^ | -^1^ |
|  |  |  | Against | 184 | 12.2% | (7.5 - 19.2%) |
|  |  |  | Don't know | 8 | -^1^ | -^1^ |
|  |  | Medium | In favor | 188 | 37.8% | (29.4 - 47.1%) |
|  |  |  | Depends | 4 | -^1^ | -^1^ |
|  |  |  | Against | 413 | 17.0% | (12.3 - 23.1%) |
|  |  |  | Don't know | 14 | -^1^ | -^1^ |
|  |  | High | In favor | 528 | 42.9% | (36.6 - 49.4%) |
|  |  |  | Depends | 32 | 21.8% | (10.7 - 39.4%) |
|  |  |  | Against | 1462 | 16.0% | (13.1 - 19.4%) |
|  |  |  | Don't know | 33 | 33.9% | (14.4 - 61.0%) |
| Burkina Faso | 2010 | Low | In favor | 359 | 66.8% | (60.3 - 72.7%) |
|  |  |  | Depends | 33 | 45.9% | (25.7 - 67.6%) |
|  |  |  | Against | 3509 | 14.8% | (13.1 - 16.6%) |
|  |  |  | Don't know | 4 | -^1^ | -^1^ |
|  |  | Medium | In favor | 389 | 53.4% | (47.2 - 59.6%) |
|  |  |  | Depends | 26 | 29.6% | (12.8 - 54.6%) |
|  |  |  | Against | 3907 | 11.0% | (9.7 - 12.5%) |
|  |  |  | Don't know | 8 | -^1^ | -^1^ |
|  |  | High | In favor | 73 | 37.9% | (26.0 - 51.3%) |
|  |  |  | Depends | 6 | -^1^ | -^1^ |
|  |  |  | Against | 965 | 5.5% | (4.1 - 7.4%) |
|  |  |  | Don't know | 1 | -^1^ | -^1^ |
| Senegal | 2017 | Low | In favor | 1278 | 71.1% | (67.4 - 74.6%) |
|  |  |  | Depends | 79 | 8.0% | (3.0 - 19.6%) |
|  |  |  | Against | 2613 | 5.3% | (4.3 - 6.5%) |
|  |  |  | Don't know | 127 | 16.8% | (10.1 - 26.6%) |
|  |  | Medium | In favor | 245 | 60.8% | (53.3 - 67.9%) |
|  |  |  | Depends | 33 | 10.7% | (4.4 - 24.1%) |
|  |  |  | Against | 1262 | 1.9% | (1.3 - 2.7%) |
|  |  |  | Don't know | 33 | 3.9% | (0.7 - 17.9%) |
|  |  | High | In favor | 144 | 34.1% | (25.9 - 43.4%) |
|  |  |  | Depends | 3 | -^1^ | -^1^ |
|  |  |  | Against | 642 | 1.4% | (0.9 - 2.3%) |
|  |  |  | Don't know | 13 | -^1^ | -^1^ |
| Chad | 2014 | Low | In favor | 855 | 34.0% | (29.8 - 38.5%) |
|  |  |  | Depends | 275 | 9.8% | (6.1 - 15.3%) |
|  |  |  | Against | 636 | 4.5% | (3.0 - 6.7%) |
|  |  |  | Don't know | 259 | 4.8% | (2.6 - 8.9%) |
|  |  | Medium | In favor | 672 | 41.5% | (36.2 - 47.0%) |
|  |  |  | Depends | 239 | 12.0% | (8.2 - 17.1%) |
|  |  |  | Against | 858 | 5.1% | (3.5 - 7.3%) |
|  |  |  | Don't know | 194 | 5.7% | (3.1 - 10.2%) |
|  |  | High | In favor | 247 | 44.0% | (37.0 - 51.3%) |
|  |  |  | Depends | 126 | 15.6% | (9.5 - 24.5%) |
|  |  |  | Against | 320 | 5.5% | (3.4 - 8.7%) |
|  |  |  | Don't know | 86 | 6.6% | (2.8 - 14.9%) |
| Côte d'Ivoire | 2011 | Low | In favor | 346 | 39.1% | (31.2 - 47.7%) |
|  |  |  | Depends | 33 | 9.3% | (2.9 - 26.0%) |
|  |  |  | Against | 1105 | 9.1% | (7.0 - 11.7%) |
|  |  |  | Don't know | 50 | 23.8% | (12.3 - 41.0%) |
|  |  | Medium | In favor | 217 | 40.7% | (33.0 - 48.9%) |
|  |  |  | Depends | 24 | -^1^ | -^1^ |
|  |  |  | Against | 1042 | 8.4% | (6.5 - 10.8%) |
|  |  |  | Don't know | 32 | 2.6% | (0.3 - 18.4%) |
|  |  | High | In favor | 136 | 36.5% | (27.7 - 46.2%) |
|  |  |  | Depends | 10 | -^1^ | -^1^ |
|  |  |  | Against | 610 | 4.5% | (2.8 - 7.0%) |
|  |  |  | Don't know | 9 | -^1^ | -^1^ |
| Kenya | 2014 | Low | In favor | 185 | 32.6% | (23.9 - 42.7%) |
|  |  |  | Depends | 7 | -^1^ | -^1^ |
|  |  |  | Against | 476 | 1.8% | (0.7 - 4.9%) |
|  |  |  | Don't know | 1 | -^1^ | -^1^ |
|  |  | Medium | In favor | 263 | 31.5% | (25.4 - 38.2%) |
|  |  |  | Depends | 24 | -^1^ | -^1^ |
|  |  |  | Against | 1773 | 1.1% | (0.7 - 1.7%) |
|  |  |  | Don't know | 8 | -^1^ | -^1^ |
|  |  | High | In favor | 454 | 29.8% | (23.2 - 37.4%) |
|  |  |  | Depends | 46 | 11.4% | (5.0 - 23.9%) |
|  |  |  | Against | 2745 | 1.4% | (1.1 - 1.9%) |
|  |  |  | Don't know | 12 | -^1^ | -^1^ |
| Tanzania | 2015 | Low | In favor | 59 | 1.0% | (0.1 - 8.4%) |
|  |  |  | Depends | 10 | -^1^ | -^1^ |
|  |  |  | Against | 615 | 0.1% | (0.0 - 0.7%) |
|  |  |  | Don't know | 13 | -^1^ | -^1^ |
|  |  | Medium | In favor | 63 | 11.9% | (5.5 - 23.9%) |
|  |  |  | Depends | 18 | -^1^ | -^1^ |
|  |  |  | Against | 2000 | 0.4% | (0.2 - 0.8%) |
|  |  |  | Don't know | 30 | 0.0% | -^2^ |
|  |  | High | In favor | 22 | -^1^ | -^1^ |
|  |  |  | Depends | 11 | -^1^ | -^1^ |
|  |  |  | Against | 1478 | 0.3% | (0.1 - 0.7%) |
|  |  |  | Don't know | 17 | -^1^ | -^1^ |
| Togo | 2013 | Low | In favor | 19 | -^1^ | -^1^ |
|  |  |  | Depends | 9 | -^1^ | -^1^ |
|  |  |  | Against | 733 | 0.3% | (0.1 - 1.1%) |
|  |  |  | Don't know | 3 | -^1^ | -^1^ |
|  |  | Medium | In favor | 26 | 6.9% | (1.4 - 28.0%) |
|  |  |  | Depends | 33 | 5.1% | (1.5 - 15.8%) |
|  |  |  | Against | 1408 | 0.2% | (0.1 - 0.5%) |
|  |  |  | Don't know | 11 | -^1^ | -^1^ |
|  |  | High | In favor | 10 | -^1^ | -^1^ |
|  |  |  | Depends | 30 | 0.0% | -^2^ |
|  |  |  | Against | 723 | 0.2% | (0.1 - 0.9%) |
|  |  |  | Don't know | 16 | -^1^ | -^1^ |
| Benin | 2011 | Low | In favor | 35 | 12.0% | (3.1 - 36.8%) |
|  |  |  | Depends | 39 | 0.0% | -^2^ |
|  |  |  | Against | 1141 | 0.2% | (0.1 - 0.7%) |
|  |  |  | Don't know | 35 | 0.0% | -^2^ |
|  |  | Medium | In favor | 71 | 1.3% | (0.1 - 10.5%) |
|  |  |  | Depends | 130 | 0.0% | -^2^ |
|  |  |  | Against | 1496 | 0.4% | (0.1 - 0.9%) |
|  |  |  | Don't know | 29 | 0.0% | -^2^ |
|  |  | High | In favor | 36 | 8.4% | (1.2 - 40.1%) |
|  |  |  | Depends | 87 | 0.0% | -^2^ |
|  |  |  | Against | 2204 | 0.2% | (0.1 - 0.5%) |
|  |  |  | Don't know | 39 | 1.9% | (0.2 - 17.2%) |

^1^ The proportion was not calculated for subgroups with less than 25 women

^2^ The confidence interval was not calculated if there were no women with at least one daughter who had undergone FGM/C in the subgroup

Table S10. Double stratification of the daughters’ FGM/C according to attitudes towards female genital mutilation/cutting and women’s empowerment (attitude towards violence)

| Country | Year | Women's empowerment (attitude towards violence) | Women's opinion on the continuation of FGM/C | n | Proportion of married/in union mothers with at least one daughter who had undergone FGM/C | Confidence interval (95%) |
| --- | --- | --- | --- | --- | --- | --- |
| Mali | 2018 | Low | In favor | 1197 | 85.8% | (83.2 - 88.1%) |
|  |  |  | Depends | 38 | 69.5% | (48.1 - 84.8%) |
|  |  |  | Against | 253 | 49.1% | (40.5 - 57.7%) |
|  |  |  | Don't know | 20 | -^1^ | -^1^ |
|  |  | Medium | In favor | 417 | 88.5% | (84.2 - 91.7%) |
|  |  |  | Depends | 31 | 74.7% | (56.3 - 87.2%) |
|  |  |  | Against | 129 | 44.9% | (33.9 - 56.5%) |
|  |  |  | Don't know | 11 | -^1^ | -^1^ |
|  |  | High | In favor | 312 | 85.6% | (80.6 - 89.5%) |
|  |  |  | Depends | 17 | -^1^ | -^1^ |
|  |  |  | Against | 126 | 49.0% | (37.6 - 60.6%) |
|  |  |  | Don't know | 13 | -^1^ | -^1^ |
| Guinea | 2018 | Low | In favor | 2226 | 56.7% | (53.8 - 59.6%) |
|  |  |  | Depends | 102 | 50.5% | (41.2 - 59.8%) |
|  |  |  | Against | 458 | 31.8% | (27.4 - 36.6%) |
|  |  |  | Don't know | 53 | 35.4% | (23.3 - 49.8%) |
|  |  | Medium | In favor | 550 | 49.0% | (43.4 - 54.6%) |
|  |  |  | Depends | 63 | 51.1% | (37.7 - 64.4%) |
|  |  |  | Against | 210 | 18.9% | (13.9 - 25.0%) |
|  |  |  | Don't know | 50 | 34.2% | (20.4 - 51.3%) |
|  |  | High | In favor | 869 | 55.0% | (51.1 - 58.8%) |
|  |  |  | Depends | 46 | 54.8% | (39.1 - 69.6%) |
|  |  |  | Against | 392 | 21.0% | (16.1 - 26.8%) |
|  |  |  | Don't know | 36 | 49.5% | (25.6 - 73.7%) |
| Nigeria | 2018 | Low | In favor | 660 | 66.3% | (61.2 - 71.0%) |
|  |  |  | Depends | 86 | 30.6% | (20.7 - 42.7%) |
|  |  |  | Against | 869 | 17.6% | (14.6 - 21.0%) |
|  |  |  | Don't know | 8 | -^1^ | -^1^ |
|  |  | Medium | In favor | 205 | 75.3% | (67.0 - 82.0%) |
|  |  |  | Depends | 73 | 31.7% | (21.2 - 44.4%) |
|  |  |  | Against | 404 | 10.8% | (8.1 - 14.2%) |
|  |  |  | Don't know | 5 | -^1^ | -^1^ |
|  |  | High | In favor | 1143 | 84.0% | (80.7 - 86.9%) |
|  |  |  | Depends | 445 | 33.8% | (28.4 - 39.6%) |
|  |  |  | Against | 3559 | 9.2% | (8.0 - 10.6%) |
|  |  |  | Don't know | 60 | 17.0% | (9.3 - 29.0%) |
| Ethiopia | 2016 | Low | In favor | 417 | 35.7% | (29.5 - 42.5%) |
|  |  |  | Depends | 22 | -^1^ | -^1^ |
|  |  |  | Against | 832 | 16.7% | (13.4 - 20.5%) |
|  |  |  | Don't know | 28 | 38.4% | (20.3 - 60.3%) |
|  |  | Medium | In favor | 144 | 52.5% | (38.3 - 66.3%) |
|  |  |  | Depends | 6 | -^1^ | -^1^ |
|  |  |  | Against | 380 | 18.6% | (13.5 - 25.1%) |
|  |  |  | Don't know | 10 | -^1^ | -^1^ |
|  |  | High | In favor | 262 | 40.2% | (29.7 - 51.8%) |
|  |  |  | Depends | 13 | -^1^ | -^1^ |
|  |  |  | Against | 847 | 13.1% | (10.1 - 17.0%) |
|  |  |  | Don't know | 17 | -^1^ | -^1^ |
| Burkina Faso | 2010 | Low | In favor | 322 | 60.6% | (54.0 - 66.9%) |
|  |  |  | Depends | 31 | 43.3% | (19.9 - 70.1%) |
|  |  |  | Against | 1989 | 15.1% | (13.1 - 17.3%) |
|  |  |  | Don't know | 3 | -^1^ | -^1^ |
|  |  | Medium | In favor | 194 | 46.3% | (37.6 - 55.3%) |
|  |  |  | Depends | 13 | -^1^ | -^1^ |
|  |  |  | Against | 1757 | 11.0% | (9.3 - 12.9%) |
|  |  |  | Don't know | 6 | -^1^ | -^1^ |
|  |  | High | In favor | 305 | 62.0% | (56.3 - 67.3%) |
|  |  |  | Depends | 21 | -^1^ | -^1^ |
|  |  |  | Against | 4635 | 10.8% | (9.6 - 12.2%) |
|  |  |  | Don't know | 4 | -^1^ | -^1^ |
| Senegal | 2017 | Low | In favor | 968 | 67.0% | (62.3 - 71.4%) |
|  |  |  | Depends | 75 | 11.0% | (4.7 - 23.8%) |
|  |  |  | Against | 1999 | 6.2% | (5.0 - 7.7%) |
|  |  |  | Don't know | 114 | 17.1% | (9.7 - 28.4%) |
|  |  | Medium | In favor | 170 | 71.0% | (62.3 - 78.4%) |
|  |  |  | Depends | 9 | -^1^ | -^1^ |
|  |  |  | Against | 520 | 2.8% | (1.7 - 4.4%) |
|  |  |  | Don't know | 21 | -^1^ | -^1^ |
|  |  | High | In favor | 529 | 61.1% | (55.2 - 66.6%) |
|  |  |  | Depends | 31 | 10.0% | (3.4 - 26.1%) |
|  |  |  | Against | 1998 | 1.9% | (1.4 - 2.5%) |
|  |  |  | Don't know | 38 | 5.1% | (1.1 - 19.8%) |
| Chad | 2014 | Low | In favor | 883 | 38.2% | (34.1 - 42.4%) |
|  |  |  | Depends | 285 | 8.4% | (5.3 - 12.9%) |
|  |  |  | Against | 982 | 4.0% | (2.8 - 5.5%) |
|  |  |  | Don't know | 314 | 5.2% | (3.2 - 8.4%) |
|  |  | Medium | In favor | 325 | 40.0% | (33.2 - 47.2%) |
|  |  |  | Depends | 168 | 18.7% | (11.5 - 28.8%) |
|  |  |  | Against | 432 | 6.6% | (4.2 - 10.2%) |
|  |  |  | Don't know | 129 | 6.3% | (3.0 - 12.6%) |
|  |  | High | In favor | 566 | 37.2% | (30.5 - 44.5%) |
|  |  |  | Depends | 187 | 11.6% | (7.6 - 17.5%) |
|  |  |  | Against | 400 | 6.4% | (4.2 - 9.7%) |
|  |  |  | Don't know | 96 | 5.2% | (2.0 - 12.8%) |
| Côte d'Ivoire | 2011 | Low | In favor | 289 | 37.1% | (28.7 - 46.4%) |
|  |  |  | Depends | 29 | 23.7% | (11.3 - 43.1%) |
|  |  |  | Against | 804 | 10.6% | (8.1 - 13.8%) |
|  |  |  | Don't know | 36 | 17.6% | (5.0 - 46.4%) |
|  |  | Medium | In favor | 137 | 33.1% | (24.4 - 43.1%) |
|  |  |  | Depends | 8 | -^1^ | -^1^ |
|  |  |  | Against | 538 | 6.8% | (4.3 - 10.4%) |
|  |  |  | Don't know | 16 | -^1^ | -^1^ |
|  |  | High | In favor | 273 | 43.6% | (35.5 - 52.0%) |
|  |  |  | Depends | 30 | 15.5% | (6.4 - 32.9%) |
|  |  |  | Against | 1415 | 6.6% | (5.1 - 8.6%) |
|  |  |  | Don't know | 39 | 18.2% | (9.5 - 32.1%) |
| Kenya | 2014 | Low | In favor | 328 | 28.7% | (21.8 - 36.7%) |
|  |  |  | Depends | 23 | -^1^ | -^1^ |
|  |  |  | Against | 1084 | 1.8% | (1.1 - 2.7%) |
|  |  |  | Don't know | 7 | -^1^ | -^1^ |
|  |  | Medium | In favor | 249 | 39.3% | (29.9 - 49.4%) |
|  |  |  | Depends | 15 | -^1^ | -^1^ |
|  |  |  | Against | 1368 | 1.5% | (1.0 - 2.3%) |
|  |  |  | Don't know | 4 | -^1^ | -^1^ |
|  |  | High | In favor | 325 | 26.8% | (21.4 - 33.0%) |
|  |  |  | Depends | 39 | 12.5% | (5.4 - 26.5%) |
|  |  |  | Against | 2542 | 1.1% | (0.7 - 1.9%) |
|  |  |  | Don't know | 10 | -^1^ | -^1^ |
| Tanzania | 2015 | Low | In favor | 84 | 4.2% | (1.3 - 13.1%) |
|  |  |  | Depends | 12 | -^1^ | -^1^ |
|  |  |  | Against | 1542 | 0.5% | (0.2 - 1.0%) |
|  |  |  | Don't know | 23 | -^1^ | -^1^ |
|  |  | Medium | In favor | 22 | -^1^ | -^1^ |
|  |  |  | Depends | 7 | -^1^ | -^1^ |
|  |  |  | Against | 819 | 0.2% | (0.1 - 0.9%) |
|  |  |  | Don't know | 10 | -^1^ | -^1^ |
|  |  | High | In favor | 38 | 13.9% | (5.3 - 31.8%) |
|  |  |  | Depends | 20 | -^1^ | -^1^ |
|  |  |  | Against | 1732 | 0.2% | (0.1 - 0.5%) |
|  |  |  | Don't know | 27 | 0.0% | -^2^ |
| Togo | 2013 | Low | In favor | 27 | 6.5% | (1.3 - 26.7%) |
|  |  |  | Depends | 31 | 0.0% | - |
|  |  |  | Against | 643 | 0.4% | (0.1 - 1.2%) |
|  |  |  | Don't know | 11 | -^1^ | -^1^ |
|  |  | Medium | In favor | 11 | -^1^ | -^1^ |
|  |  |  | Depends | 22 | -^1^ | -^1^ |
|  |  |  | Against | 468 | 0.2% | (0.0 - 1.2%) |
|  |  |  | Don't know | 12 | -^1^ | -^1^ |
|  |  | High | In favor | 17 | -^1^ | -^1^ |
|  |  |  | Depends | 19 | -^1^ | -^1^ |
|  |  |  | Against | 1753 | 0.2% | (0.1 - 0.5%) |
|  |  |  | Don't know | 7 | -^1^ | -^1^ |
| Benin | 2011 | Low | In favor | 17 | -^1^ | -^1^ |
|  |  |  | Depends | 20 | -^1^ | -^1^ |
|  |  |  | Against | 375 | 0.6% | (0.1 - 2.4%) |
|  |  |  | Don't know | 14 | -^1^ | -^1^ |
|  |  | Medium | In favor | 11 | -^1^ | -^1^ |
|  |  |  | Depends | 26 | 0.0% | -^2^ |
|  |  |  | Against | 563 | 0.7% | (0.2 - 2.5%) |
|  |  |  | Don't know | 12 | -^1^ | -^1^ |
|  |  | High | In favor | 114 | 4.0% | (1.4 - 11.1%) |
|  |  |  | Depends | 210 | 0.0% | -^2^ |
|  |  |  | Against | 3903 | 0.2% | (0.1 - 0.4%) |
|  |  |  | Don't know | 77 | 0.9% | (0.1 - 7.1%) |

^1^ The proportion was not calculated for subgroups with less than 25 women

^2^ The confidence interval was not calculated if there were no women with at least one daughter who had undergone FGM/C in the subgroup
